# Pharmacogenomic Biomarkers in Docetaxel Treatment of Prostate Cancer: From Discovery to Implementation

**DOI:** 10.3390/genes10080599

**Published:** 2019-08-08

**Authors:** Reka Varnai, Leena M. Koskinen, Laura E. Mäntylä, Istvan Szabo, Liesel M. FitzGerald, Csilla Sipeky

**Affiliations:** 1 Department of Primary Health Care, University of Pécs, Rákóczi u 2, H-7623 Pécs, Hungary; 2 Faculty of Health Sciences, Doctoral School of Health Sciences, University of Pécs, Vörösmarty u 4, H-7621 Pécs, Hungary; 3 Institute of Biomedicine, University of Turku, Kiinamyllynkatu 10, FI-20520 Turku, Finland; 4 Institute of Sport Sciences and Physical Education, University of Pécs, Ifjúság útja 6, H-7624 Pécs, Hungary; 5 Faculty of Sciences, Doctoral School of Biology and Sportbiology, University of Pécs, Ifjúság útja 6, H-7624 Pécs, Hungary; 6 Menzies Institute for Medical Research, University of Tasmania, Hobart, Tasmania 7000, Australia

**Keywords:** castration resistant prostate cancer, docetaxel, pharmacogenomic biomarker, personalised treatment

## Abstract

Prostate cancer is the fifth leading cause of male cancer death worldwide. Although docetaxel chemotherapy has been used for more than fifteen years to treat metastatic castration resistant prostate cancer, the high inter-individual variability of treatment efficacy and toxicity is still not well understood. Since prostate cancer has a high heritability, inherited biomarkers of the genomic signature may be appropriate tools to guide treatment. In this review, we provide an extensive overview and discuss the current state of the art of pharmacogenomic biomarkers modulating docetaxel treatment of prostate cancer. This includes (1) research studies with a focus on germline genomic biomarkers, (2) clinical trials including a range of genetic signatures, and (3) their implementation in treatment guidelines. Based on this work, we suggest that one of the most promising approaches to improve clinical predictive capacity of pharmacogenomic biomarkers in docetaxel treatment of prostate cancer is the use of compound, multigene pharmacogenomic panels defined by specific clinical outcome measures. In conclusion, we discuss the challenges of integrating prostate cancer pharmacogenomic biomarkers into the clinic and the strategies that can be employed to allow a more comprehensive, evidence-based approach to facilitate their clinical integration. Expanding the integration of pharmacogenetic markers in prostate cancer treatment procedures will enhance precision medicine and ultimately improve patient outcomes.

## 1. Introduction

Prostate cancer (PC) remains the second most common cancer in men, and one of the leading causes of death among Western males [1]. This is due to the fact that treatment of metastatic prostate cancer (mPC) is becoming increasingly challenging [2,3]. Docetaxel chemotherapy was approved 15 years ago to treat metastatic castration-resistant prostate cancer (mCRPC), and is now standard care for this stage of disease [2]. Although other drugs have since been developed, some of which are administered in combination regimens with docetaxel, docetaxel remains the main choice of chemotherapeutic agent [4].

Significant progress has been made in genetic biomarker-based treatment of several cancer types [5,6]; however, personalized treatment of PC is lagging behind. Also, it is increasingly evident that the wide variability in treatment response, toxicity, and disease progression between PC patients is due to the genetic heterogeneity of the disease. Therefore, underlying genetic variations are potentially eligible biomarkers for targeted therapy, or to predict drug response and adverse side effects [7]. Treatment-associated, germline genomic biomarkers have several advantages: they are static, can be easily determined, and are robust predictors of drug response/resistance and toxicity. Biomarkers, including somatic genomic alterations, structural variants (e.g., gene fusions, gene rearrangements), splice variants, miRNAs, and differential gene expression, and methylation markers have also been shown to modulate docetaxel treatment of PC [8].

The focus of this review is to discuss the current state-of-the-art pharmacogenomic biomarkers modulating docetaxel treatment of PC. The review includes research studies focusing on germline genomic biomarkers, clinical trials designed to incorporate all type of biomarkers, and finally, the implementation of biomarkers in treatment guidelines.

## 2. Docetaxel in Prostate Cancer Treatment

Docetaxel is a taxane, a chemotherapeutic agent that produces antitumour activity. It has been previously approved for the treatment of breast cancer and non-small-cell lung cancer, and was approved by the United States Food and Drug Administration on May 19, 2004 for use in combination with prednisone for the treatment of metastatic, androgen-independent prostate cancer (AIPC)/hormone-refractory prostate cancer (HRPC) [9,10]. Docetaxel is a semi-synthetic, second-generation taxane derived from a compound found in the European yew tree (*Taxus baccata*). Docetaxel displays potent and broad antineoplastic properties. It binds to and stabilizes tubulin, thereby inhibiting microtubule disassembly, which results in cell-cycle arrest at the G2/M phase and cell death. This agent also inhibits pro-angiogenic factors, such as vascular endothelial growth factor (VEGF), and displays immunomodulatory and pro-inflammatory properties by inducing various mediators of the inflammatory response. Docetaxel has been studied for use as a radiation-sensitizing agent as well [11].

The pharmacodynamics and pharmacokinetics of docetaxel are extremely complex and have been the subject of intensive investigation. Docetaxel is metabolized both by CYP3A4 and CYP3A5 [12]. Docetaxel is the substrate for the ATP-binding, cassette multidrug transporters ABCB1, ABCG2, ABCC1 and ABCC2. However, SLCO1B3 was identified as the most efficient influx transporter for docetaxel [13].

Unfortunately, most patients develop resistance to docetaxel. Mechanisms of resistance to chemotherapy include tubulin alterations, increased expression of multidrug resistance genes, *TMPRSS2*–*ERG* fusion genes, kinesins, cytokines, components of other signaling pathways, and epithelial–mesenchymal transition [14].

It is important to note that docetaxel has no PC treatment-guiding pharmacogenomic biomarker included on the drug label, based on the information available from the U.S. Food and Drug Administration (FDA) [15] and the European Medicines Agency (EMA) [16].

## 3. Germline Genomic Biomarkers in Research Studies for Prostate Cancer Treatment with Docetaxel

Clinical research studies have investigated the genomic biomarkers of docetaxel monotherapy; however, combination therapies with distinct mechanisms of action represent a more effective strategy. Combination therapies are thought to exert cancer-killing functions through either concomitant targeting of multiple pro-cancer factors or more effective inhibition of a single pathway [17]. The exact mechanisms by which these combinations can overcome drug resistance have yet to be fully understood [17].

Studies of germline genomic biomarkers affecting individual differences in docetaxel monotherapy (I) and combination treatment (II) of PC published between 2006 and 2018 are summarized in chronological order in Table 1.

### 3.1. Docetaxel Monotherapy

Tran et al. [18] studied the pharmacokinetics of docetaxel and concluded that *CYP3A4* (rs2740574) and *CYP3A5* (rs776746) polymorphisms are associated with enhanced docetaxel clearance. Therefore, patients carrying the CYP3A4*1B allele may be underexposed to the treatment. Furthermore, GSTP1*A/*B (rs1695) and *MDR1* 3435TT (rs1045642) carriers are linked to excessive hematologic febrile neutropenia toxicity [18]. A second study has also suggested that variants in *ABCC2* (rs12762549) and *SLCO1B3* (rs11045585) may predict the risk of leukopenia/neutropenia induced by docetaxel chemotherapy [19]. However, in a study of 64 U.S. cancer patients who received a single cycle of 75 mg/m^2^ of docetaxel monotherapy, the *ABCC2* variant rs12762549 showed a trend towards reduced docetaxel clearance, but no association with neutropenia was observed [20].

A case report of a 55-year-old male treated with docetaxel after a radical prostatectomy has suggested that the *CYP1B1* gene may play a role in modulating docetaxel activity [21]. The rs1056836 and rs1800440 *CYP1B1* missense variants were linked to better overall survival (OS) of the patient, who remained disease free until publication of the article (two years). The *CYP1B1* isoforms of Leu432 and Ser453 are characterized by inferior catalytic activity, and while docetaxel is not metabolized by *CYP1B1*, its low activity may favorably influence docetaxel sensitivity by impaired estrogen metabolite production, which in turn could interfere with binding of the drug to tubulin [21].

Sobek and colleagues studied variants of the ABCG2 transporter protein, which effluxes folate, dihydrotestosterone, and chemotherapeutic drugs, among other molecules, out of cells [22]. In in vitro experiments using HEK293 cells (as exogenous *ABCG2* expression in PC cell lines led to selective disadvantage), the rs2231142 (Q141K) variant was observed to efflux less folate. This variant makes the cells more sensitive to docetaxel treatment compared to the wild-type ABCG2. Based on these findings, the authors conclude that the Q141K variant predisposes the cells to less efficient docetaxel efflux, leading to increased intracellular docetaxel levels and thus increased docetaxel sensitivity. The effect of decreased folate efflux was also observed in PC patients carrying the Q141K variant; serum folate levels were significantly lower compared to patients carrying wild-type ABCG2. The authors suggested that increased intra-tumoral folate levels enhance cancer cell proliferation, which may explain why patients with the Q141K variant had a significantly shorter time to prostate-specific antigen (PSA) recurrence after a prostatectomy. The authors concluded that PC patients with the Q141K variant may have a better response to docetaxel, and they may respond differently to treatments that aim to inhibit the efflux of chemotherapeutic agents [22].

### 3.2. Docetaxel Combination Therapies

#### 3.2.1. Docetaxel and Vinorelbine or Estramustine Phosphate

The first investigation of combination therapies was done in 2006. Here, the role of the *ABCG2* variant rs2231142 (421C>A; Q141K) in treatment response has been studied in HRPC patients treated with docetaxel and vinorelbine/estamustine phosphate [23]. There was a significant association between survival beyond 15 months and the *ABCG2* rs2231142 polymorphism. The increased survival seen in individuals with an *ABCG2* rs2231142 polymorphism may suggest a less functional drug efflux pump, leading to increased intracellular (intra-tumoral) docetaxel concentration and improved cytotoxic activity, lower transporter expression, and improved survival. This variant may therefore be an important predictor of response and survival in HRPC patients treated with docetaxel-based chemotherapy. The companion pharmacogenetic study assessed germ-line polymorphisms in genes known to play important roles in chemotherapy drug transport, metabolism, and mechanism of action. The effect of *ABCG2* polymorphisms on docetaxel pharmacokinetics is unknown [23].

#### 3.2.2. Docetaxel and Estramustin, Thalidomide, and Prednisone

The role of *CYP1B1* variation in treatment response has also been investigated in AIPC patients receiving docetaxel-based combination therapies with estramustin, thalidomide, and prednisone [24]. Individuals carrying two copies of the CYP1B1*3 (rs1056836) variant had a poor prognosis compared to individuals carrying at least one copy of the CYP1B1*1 ancestral allele. The association between CYP1B1*3 and response to therapy was not observed in comparable subjects receiving non-taxane-based therapy. The systemic clearance of docetaxel was also unrelated to *CYP1B1* genotype status, indicating that the association of CYP1B1*3 with clinical response (CR) is not due to docetaxel metabolism. This pilot study provides evidence that CYP1B1*3 may be an important marker for estimating docetaxel efficacy in patients with AIPC. This link is likely associated with CYP1B1*3 genotype-dependent estrogen metabolism. Specifically, that CYP1B1-generated estrogen metabolites may bind to tubulin [25], and potentially could interfere with docetaxel-mediated tubulin stabilization. In addition, estrogen metabolites may also react with docetaxel and structurally alter the drug [24].

#### 3.2.3. Docetaxel and Thalidomide

Docetaxel therapy in combination with thalidomide has led to several pharmacogenomic findings. Thalidomide is suggested to play a role in inflammation, immunomodulation, and anti-angiogenesis, and thus influences disease progression [26]. A study by Sissung et al. investigated the association of *ABCB1* 1236C>T (rs1128503), 2677 G>T/A (rs2032582), and 3435 C>T (rs1045642) polymorphisms and treatment efficacy, measured by survival after treatment or peripherial neuropathy in AIPC patients treated with docetaxel alone (*n* = 23) or docetaxel and thalidomide (*n* = 50) [27]. While the *ABCB1* 1236C-2677G-3435C ancestral haplotype was associated with improved OS in docetaxel treated patients, the *ABCB1* 2677T-3435T variant haplotype was significantly associated with shorter median OS in patients treated with both docetaxel and thalidomide. Among both treatment arms together, individuals carrying the 2677GG ancestral genotype had a significantly longer time to neuropathy. Finally, there was a strong trend toward patients carrying the 2677TT-3435TT diplotype having higher grades of neutropenia. Interestingly, none of the variants associated with OS or toxicity had a significant effect on docetaxel pharmacokinetics [27]. These results suggest that variant alleles associated with lowered *ABCB1* expression and altered function result in a clinical phenotype of reduced docetaxel efficacy and increased toxicity (TOX) in men with AIPC. It is possible that expression of *ABCB1* outside of the liver is responsible for these findings, as polymorphic *ABCB1* variants can modulate the exposure of *ABCB1* substrates in tumor cells where this gene is highly up-regulated. It is also notable that efficacy is decreased while TOX is increased in patients carrying variant alleles [27].

Additional genetic polymorphisms have been analysed for associations with clinical response (CR) and TOX in a study of CRPC patients receiving either docetaxel and thalidomide or docetaxel alone [28]. *PPAR-δ* variants rs6922548, rs2016520, rs1883322, rs3734254, and rs7769719, as well as the *SULT1C2* variant rs1402467 were all observed to be associated with CR. Several variants in the *CHST3* gene were linked to CR exclusively (rs4148943, rs4148947, rs12418, and rs730720), while others were liked to both CR and TOX (rs4148950, rs1871450, and rs4148945). Variants in *SPG7* (rs2292954, rs12960), CYP2D6 (CYP2D6*19), *NAT2* (rs1799931), *ABCC6* (rs2238472), *ATP7A* (rs2227291), *CYP4B1* (rs4646487), and *SLC10A2* (rs2301159) were associated exclusively with TOX. These data revealed that polymorphisms in three genes (*PPAR-δ*, *SULT1C2*, and *CHST3*) were associated with clinical outcome measure of OS, whereas polymorphisms in eight genes (*SPG7, CHST3, CYP2D6, NAT2, ABCC6, ATP7A, CYP4B1*, and *SLC10A2*) were associated with TOX. Although all of these genes may be related to drug metabolism directly, and thus could be related to pharmacokinetics, they also participate in pathways that may affect drug action and could therefore be involved in pharmacodynamic interactions as well. Differences between the two treatment arms were seen exclusively in the *PPARδ* gene, where strong relationships with *PPARδ* single nucleotide polymorphisms (SNPs) were observed in only those patients who received both docetaxel and thalidomide, but not docetaxel alone. This shows that allelic variation in *PPARδ* may influence the therapeutic efficacy of the anti-angiogenesis agent thalidomide [28].

As genetic variability in liver enzymes is often linked to interindividual variation in liver metabolism, Sissung et al. hypothesised that certain variants and genes in these pathways may be behind the risk and prognosis of CRPC [29]. Patients treated with docetaxel and thalidomide and who carried variants in *ABCB11* (rs7602171 GA/AA), *ABCB4* (rs2302387 CT), *ABCC5* (rs939339 AG), and *SLC5A6* (rs1395 GA/AA) had poor OS compared to those carrying only wild-type alleles, whereas the *GSTP1* rs1799811 CT genotype was associated with prolonged OS. Of considerable interest are several associations between CRPC prognosis and protein transporters that regulate bodily sterol and fatty acid deposition. In this small pilot study, there was suggestive evidence that SNPs in bile acid and fat catabolism genes may be related to CRPC OS. No evidence was found that any of the aforementioned SNPs were related to risk of developing CRPC [29].

#### 3.2.4. Docetaxel and Prednisone

*CYP1B1* variation has also been studied in relation to its role in modulating docetaxel treatment response when combined with prednisone [30]. Patients carrying the *CYP1B1-*432ValVal (rs1056836, corresponding to 4326GG) genotype experienced a significantly lower response rate, as well as shorter progression-free survival (PFS) and OS, and its prognostic significance for OS was confirmed. In contrast, no correlations were observed between both the *CYP1B1* C142G (rs10012) or *CYP1B1* A4390G (rs1800440) polymorphisms and clinical outcome in CRPC patients treated with docetaxel and prednisone. In summary, the *CYP1B1* 4326GG polymorphism was linked to docetaxel CR, and may represent a potential new marker for treatment optimization [30].

#### 3.2.5. Docetaxel and Estramustine, Thalidomide, and Ketoconazole

To explore the role of variants in the estrogen pathway and treatment response in a clinical trial setting, CRPC patients treated with docetaxel monotherapy, or different combinations of docetaxel with estramustine, thalidomide, and ketoconazole were genotyped for polymorphisms in estrogen synthesis (*CYP19* rs700519) and estrogen target (*ERα* rs2234693, rs9340799) genes [31]. Patients carrying two copies of *ERα* polymorphisms had shorter progression-free survival (PFS) on docetaxel than other patients. When the analysis was limited to non-obese patients, the relationship between the *ERα* rs9340799 polymorphism and PFS improved. These results supported the hypothesis that reactive estrogen species cause genotoxicity, and may interfere with docetaxel-mediated tubulin polymerization, resulting in shortened survival in men with CRPC. The *CYP19* variant was moderately associated with the duration of survival after docetaxel therapy in patients who were greater than 70 years old. Both *ERα* polymorphisms were also associated with an increase in CRPC risk, and the association with *ERα* variant rs2234693 also improved in those men who were greater than 70 years old. This study demonstrates that estrogen-related genetic variation affects docetaxel CR, and that this relationship is dependent on age and body type in men with CRPC. Moreover, this study suggests that *ERα* polymorphisms confer the risk of developing CRPC, especially in men under 70 years of age [31].

#### 3.2.6. Docetaxel, Prednisone, and Metronomic Cyclophosphamide

Since VEGF is thought to play an important role in angiogenesis and tumor proliferation, a study of the *VEGF* gene in mCRPC patients treated with a combination of docetaxel, prednisone, and metronomic cyclophosphamide was done [32]. The authors observed significantly longer PFS in patients carrying the *VEGF* rs1570360 AG/GG genotypes. Notably, the AA genotype was associated with reduced *VEGF* transcription, suggesting that tumors with the *VEGF* 21154 AG/GG genetic background may produce higher VEGF-A levels after the administration of standard chemotherapy. The authors suggest that *VEGF* and bFGF plasma levels at the end of the first cycle of chemotherapy and *VEGF* genotyping may be used to predict which patients will have greater PFS from this particular combination of therapies [32].

#### 3.2.7. Docetaxel and Atrasentan

Finally, the role of variation in the α-1 acid glycoprotein *(AAG)* gene has been explored in PC patients receiving combination intravenous docetaxel and oral atrasentan therapy [33]. The results suggested that the *AAG* genetic polymorphism, rs250242, may explain some inter-patient variability in docetaxel pharmacokinetics. An evaluation of the pharmacokinetics of both drugs showed that the systemic clearance of docetaxel was increased by approximately 21% when given concomitantly with atrasentan; however, atrasentan pharmacokinetics did not appear to be influenced by docetaxel administration [33].

#### 3.2.8. Docetaxel and Dexamethasone

A genome-wide association study of docetaxel treatment in combination with dexamethasone in hormone-refractory PC patients has shown that the rs875858 SNP in *VAC14* is significantly associated with increased neuropathy risk, irrespective of patient randomisation to bevacizumab or a placebo [34]. While not significant genome-wide, two additional *ATP8A2* SNPs, rs11017056 and rs1326116, showed a trend towards increased neuropathy risk. The authors recommend that *VAC14* should be prioritized for further validation to determine its role as a predictor of docetaxel-induced neuropathy and as a biomarker for treatment individualization.

**Table 1:** Research studies of germline biomarkers in docetaxel and combination treatment of prostate cancer.

Biomarker	Variant	Effect	Number of Samples/Study Method	Study Type	Country	Reference
**I. Docetaxel Monotherapy**						
CYP3A4	rs2740574 (c.−392G>A)	D (Clearance↑)	58 patients initiating chemotherapy	Interventional	France	Tran et al. [18]
CYP3A5	rs776746(c.219−237A>G)	D (Clearance↑)				
GSTP1	rs1695 (A313G, Ile105Val)	TOX				
MDR1	rs1045642 (C3435T, Ile1145Ile)	TOX				
ABCC2	rs12762549	TOX	84 patients: 28 patients with leukopenia/neutropenia vs. 56 with no TOX	Case–control	Japan	Kiyotani et al. [19]
SLCO1B3	rs11045585	TOX				
CYP1B1	rs1056836 (C1294G, Leu432Val)	OS	55-year-old male with multifocal adenocarcinoma; 75 mg/m^2^ docetaxel every three weeks for six cycles	Case report	Italy	Brandi et al. [21]
	rs1800440 (A1358G, Asn453Ser)					
ABCC2	rs12762549	D (Clearance↓)	64 patients received a single cycle of 75 mg/m^2^ docetaxel	Interventional	United States	Lewis et al. [20]
SLCO1B3	rs11045585	No effect				
ABCG2	rs2231142 (C421A, Q141K)	CR	HEK293 cells, 40 patients	In vitro, Validated in vivo	United States	Sobek et al. [22]
**II. Docetaxel Combination Therapies**						
**Docetaxel and Vinorelbine, Estramustine Phosphate**						
ABCG2	rs2231142 (C421A, Q141K)	OS	64 chemotherapy-naive patients with HRPC were randomized to (1) docetaxel (20 mg/m^2^ i.v. days 1 and 8) + vinorelbine (25 mg/m^2^ i.v. days 1 and 8) and (2) docetaxel (60–70 mg/m^2^ i.v. day 1) + estramustine phosphate (280 mg oral 3x/day, days 1–5)	Interventional	United States	Hahn et al. [23]
**Docetaxel and Estramustin, Thalidomide, Prednisone**						
CYP1B1	rs1056836 (C4326G, Leu432Val)	OS	52 patients with AIPC: (1) docetaxel (*n* = 25, 1 h i.v.,30 mg/m^2^); (2) docetaxel + estramustine + thalidomide (*n* = 20, 30 min i.v., 30 mg/m^2^) docetaxel + prednisone (*n* = 7, 1 h i.v., 75 mg/m^2^)	Observational retrospective	United States	Sissung et al. [24]
**Docetaxel and Thalidomide**						
ABCB1	rs1128503 (C1236T)	OS	AIPC patients; 50 patients with docetaxel + thalidomide; 23 patients with docetaxel;	Interventional	United States	Sissung et al. [27]
	rs2032582 (G2677T/A)	OS, TOX				
	rs1045642 (C3435T)	OS, TOX				
PPAR-δ	rs6922548	CR	74 CRPC patients: (1) CRPC patients (*n* = 25) with docetaxel (30 mg/m^2^ weekly for three weeks, followed by a one-week rest); (2) patients (*n* = 49) with docetaxel (30 mg/m^2^ weekly for three weeks followed by a one-week rest) + thalidomide (200 mg orally each day)	Interventional	United States	Deeken et al. [28]
	rs2016520	CR				
	rs1883322	CR				
	rs3734254	CR				
	rs7769719	CR				
CHST3	rs4148943	CR				
	rs4148947	CR				
	rs12418	CR				
	rs730720	CR				
	rs4148950	CR, TOX				
	rs1871450	CR, TOX				
	rs4148945	CR, TOX				
SULT1C2	rs1402467	CR				
SPG7	rs2292954	TOX				
	rs12960	TOX				
CYP2D6	*19 (2539_2542delAACT)	TOX				
NAT2	rs1799931	TOX				
ABCC6	rs2238472	TOX				
ATP7A	rs2227291	TOX				
CYP4B1	rs4646487	TOX				
SLC10A2	rs2301159	TOX				
ABCB4	rs2302387	OS	74 CRPC patients: (1) patients (*n* = 49) with docetaxel (30 mg/m^2^ weekly for three weeks followed by a one-week rest); (2) patients (*n* = 25) with docetaxel (same schedule) + thalidomide (200 mg orally each day)	Observational, retrospective	United States	Sissung et al. [29]
ABCB11	rs7602171	OS				
ABCC5	rs939336	OS				
GSTP1	rs1799811	OS				
SLC5A6	rs1395	OS				
**Docetaxel and Prednisone**						
CYP1B1	rs10012 (C142G, Arg48Gly)	No effect	60 CRPC patients: (1) docetaxel (1 h, 75 mg/m^2^ on day 1) every 21 days, or (2) docetaxel (30 mg/m^2^ weekly for five of every six weeks) + prednisone (10 mg os daily)	Interventional	Italy	Pastina et al. [30]
	rs1056836 (C4326G, Leu432Val)	CR, OS, PFS				
	rs1800440 (A4390G, Asn453Ser)	No effect				
**Docetaxel and Estramustine, Thalidomide, Ketoconazole**						
CYP19 (now CYP19A1)	rs700519 (c.C790T, R264C)	OS	111 CRPC patients: (1) *n* = 20 with estramustine, docetaxel, and thalidomide; (2) *n* = 21 with ketoconazole + docetaxel; (3) *n* = 50 with docetaxel + thalidomide; (4) *n* = 24 with docetaxel alone; 289 healthy controls	Observational, retrospective	United States	Sissung et al. [31]
ERα (now ESR1)	rs2234693	OS				
	rs9340799	OS				
**Docetaxel and Prednisone and Metronomic CTX**						
VEGF-A	rs699947 (A22578C)	PFS	41 mCRPC patients on day 1 received docetaxel (60 mg/m^2^ intravenously every three weeks, up to 12 cycles) + prednisone (10 mg/day, from day 2 continuously) + celecoxib 200 mg orally 2×/day	Interventional	Italy	Derosa et al. [32]
	rs1570360 (A21154G)	PFS				
	rs2010963 (C2634G)	PFS				
	rs3025039 (C1936T)	PFS				
**Docetaxel and Atrasentan**						
AAG	rs250242 (A4069G)	Clearance↑. No info about dosage effect.	21 PC patients; docetaxel (60–75 mg/m^2^, every 3 weeks, i.v.) + atrasentan (10 mg/day starting on day 3 of cycle 1, given continuously, oral)	Interventional	United States	Younis et al. [33]
**Docetaxel and Dexamethasone**						
ATP8A2	rs11017056	TOX	623 mCRPC Caucasian patients randomized into two arms; drugs were administered to both arms (arm 1 and arm 2): docetaxel (75 mg/m^2^ i.v., 1 h on day 1 of each 21-day cycle) + dexamethasone (8 mg oral, 12, 3, 1 h prior to docetaxel i.v.) + prednisone (5 mg oral 2×/day); (arm 1) adding bevacizumab (15 mg/kg i.v. on day 1 of each cycle), and (arm 2) adding placebo (i.v. on day 1 of each cycle)	Interventional	United States	Hertz et al. [34]
	rs1326116	TOX				
VAC14	rs875858	TOX				

SNP: single nucleotide polymorphism; mCRPC: metastatic castration resistant prostate cancer; PC: prostate cancer; HRPC: hormone resistant prostate cancer; AIPC: androgen-independent prostate cancer; i.v.: intravenous; D: dosing; TOX: toxicity; OS: overall survival; CR : clinical response; PFS: progression free survival; CTX: cyclophosphamide.

## 4. Clinical Trials of Docetaxel Treatment in Prostate Cancer Incorporating Genomic Signature

Clinical trials have been identified both from ClinicalTrials.gov [35] and from the European Union (EU) Clinical Trials Register database [36]. Only trials that included patients with PC, docetaxel as the administered treatment, and evidence of incorporation of genomic signature analyses were included in this review.

ClinicalTrials.gov and the EU Clinical Trials Register use different terminology for describing the status of a trial. On ClinicalTrials.gov, the status can be "completed”, “terminated”, “withdrawn”, “recruiting”, and “active”, as well as “not recruiting”, “not yet recruiting” or “unknown”. “Terminated” trials have stopped early, but participants have been recruited and they have received intervention, whereas “withdrawn” trials have stopped before the recruitment of participants. “Active” and “not recruiting” trials have recruited participants who are currently receiving intervention or are going through examinations, whereas “not yet recruiting” trials have not recruited any participants. Therefore, we collectively refer to the “recruiting”, “active”/”not recruiting”, and “not yet recruiting” trials as ongoing trials. In the EU Clinical Trials Register, the status of a trial can be “completed”, “prematurely ended”, or “ongoing”.

### 4.1. Biomarkers in ClinicalTrials.gov

Overall, 132 trials were found from ClinicalTrials.gov with the search algorithm described above. After removing duplicate results and irrelevant trials, the number of the remaining and analysed trials was 24.

Of note, there were fewer “completed” or “terminated” trials (Table 2) than “ongoing” clinical trials (Table 3) [37], indicating the intense translational interest in this field. The reasons for trial terminations were withdrawal of funding (NCT00503984) or low participant enrollment (NCT01253642). Four trials had been withdrawn before recruitment of patients, and two trials had unknown status (Supplementary Table S1).

The majority of trials were interventional, with only two being observational. In the group of interventional trials, the phase of the study was defined for 15 trials, most of which were in phase II [38] (Table 2 and Table 3). In the majority of interventional trials, docetaxel was explored in different settings of combination treatments. In the observational studies, docetaxel was compared to cabazitaxel and paclitaxel (Table 3), novel antineoplastic agents that interfere with microtubule function, leading to altered mitosis and cellular death [39].

The genomic biomarkers evaluated in the trials were not always precisely defined, indicating only that the target of the investigation was a gene expression profile or genes related to treatment efficacy, but not specifying further. Furthermore, the genetic analyses were inexact in many cases. Here, we summarize the “completed” or “terminated” clinical trials with output measures and the “ongoing” trials with possible future results, with special focus on the trials where the genomic profiling is specified.

Results have been published on two “completed” and two “terminated” trials (Table 2). However, the results of the completed trials did not include genomic results. In one of these trials (NCT00089609), the intervention treatment included docetaxel, prednisone, thalidomide, and bevacizumab, and the studied genes were *CYP3A4* and *CYP3A5* for docetaxel metabolism and *CYP2C19* for thalidomide metabolism. The exact genetic variants studied and their association with efficacy were not described in the results. The other “completed” trial (NCT01308567) with results aimed to investigate the pharmacogenomics of cabazitaxel, but not docetaxel; however, docetaxel was included in the intervention.

The genetic results of the two “terminated” trials seem to be more impactful. The aim of one of these, NCT00503984, was to determine whether azacitidine could reverse docetaxel resistance in mCRPC patients by decreasing methylation of the proapoptotic *GADD45A* gene [40]. The authors had previously observed that methylation of *GADD45A* in DU145 PC cells increases during docetaxel treatment and contributes to docetaxel resistance [41]. In addition, they found that azacitidine treatment decreases the methylation of *GADD45A* and restores docetaxel sensitivity in resistant PC cells. In the clinical trial, changes in *GADD45A* methylation were examined in buffy-coat DNA of patients. After azacitidine treatment, methylation significantly decreased in ten patients, increased in four patients, and in one patient could not be assessed due to a lacking sample (Phase I, 15 patients). Six of the ten patients with decreased methylation also had a concomitant decrease in the PSA level, while none of the four patients with increased methylation had a PSA response. However, the difference was not statistically significant (*p* = 0.085). The authors concluded that the addition of azacytidine could be beneficial in mCRPC patients after initial docetaxel treatment failure [40]. With regards to the second “terminated” trial (NCT01253642), only the frequency of *MAOA* (monoamine oxidase A) overexpression in tumors that have progressed during docetaxel treatment was reported. *MAOA* overexpression was observed in all investigated progressing tumors.

The focus of several ongoing clinical trials (Table 3) is treatment response to docetaxel treatment in combination with emerging new medications in tumors harbouring inactive mutations in homologous recombination (HR) genes, including *BRCA1*, *BRCA2*, and *ATM*. Five recruiting trials plan to study the effect of these genes on treatment response, where treatments including a poly-ADP ribose polymerase (PARP) inhibitor (rucaparib), a nonsteroidal antiandrogen (enzalutamide), or a chemotherapy drug (carboplatin), combined with or compared to docetaxel.

A promising recruiting trial, NCT03218826, plans to evaluate the effect of docetaxel combined with AZD8186, a novel potent small molecule, which targets the lipid kinase PI3Kβ signaling and inhibits the growth of *PTEN*-deficient prostate tumors [42].

The effect of androgen receptor (*AR*) gene alterations and splice variants on treatment response are going to be evaluated in two trials. The impact of these alterations on PSA response will be evaluated in docetaxel treatment combined with enzalutamide (NCT03700099), and on patient prognosis related to docetaxel versus cabazitaxel treatment (NCT02362620), in addition to the effect of *TMPRSS2-ERG* rearrangement and *PTEN* loss.

Only one trial (NCT03816904) plans to focus on the adverse effects of docetaxel. The aim of this trial is to investigate the association between the number of CAG triplets in the *KCNN3* gene (which codes for the SK3 calcium channel) and taxane neuropathy in patients who are receiving either docetaxel or paclitaxel. This trial is a prospective observational trial, and plans to follow patients with different types of cancer, including PC patients.

### 4.2. Biomarkers in the EU Clinical Trials Register

In addition to the ClinicalTrials.gov database, clinical trials for docetaxel chemotherapy with pharmacogenetic aspects were searched for in the EU Clinical Trials Register [36]. A total of 76 trials were found, and after removing duplicate and irrelevant search results, only four trials remained.

Of the four trials, one was “completed”, one was “terminated”, and two were “ongoing” (Table 4). Results have been published for the completed and the terminated trials, but no pharmacogenetic aspects were presented, and only one trial (EudraCT 2006-004478-29) specified which genes (*CYP2B6*, *CYP2C19*, *CYP2C9*, and *CYP3A5*) they planned to investigate. In two of the trials, descriptions of the genetic biomarker investigations were included in a sub-study (EudraCT 2013-000809-23) or in a separate study planned to be conducted later based on samples collected during the actual trial (EudraCT 2008-000701-11); however, the specific biomarkers to be studied were not provided.

Interestingly, three of the four trials were found retrospectively on ClinicalTrials.gov, but none of them was found with the search algorithm used there. The reason for this is that the pharmacogenomic aspects were not mentioned on ClinicalTrials.gov, but they were included to the EU register, albeit briefly. Notably, in one of these trials the original secondary outcome measures on ClinicalTrials.gov included the evaluation of genetic biomarkers, but this outcome measure had later been deleted from the trial description. This change had not been updated in the EU Clinical Trials Register.

## 5. Pharmacogenomic Biomarkers in Prostate Cancer Treatment Guidelines

The European Association of Urology (EAU) [43,44] and European Society for Medical Oncology (ESMO) [45] PC treatment guidelines were reviewed for any recommendations on pharmacogenetic testing before or during docetaxel treatment. In general, the ESMO guideline states that there are no predictive biomarkers to guide treatment decisions, even though there are some known prognostic biomarkers. On the other hand, the EAU guideline discusses multiple diagnostic or prognostic genetic biomarkers and their use in the clinic. These guidelines suggest that the first future application of pre-emptive genetic testing commence and involve homologous recombination deficiency genes, since these patients might benefit from treatment with PARP inhibitors [43]. However, no definite recommendation has been made.

## 6. Biomarkers with Translational Potential in Docetaxel Treatment of Prostate Cancer

Predictive pharmacogenomic biomarkers of the highest importance, with clinical implementational potential, are the ones affecting clinical response. Based on research studies on germline genomic biomarkers, we can conclude that variants in *CYP1B1, ABCG2, CHST3, PPAR-δ,* and *SULT1C2* genes have a documented impact on better clinical response to docetaxel treatment in PC (Table 5). Pre-emptive genotyping of pharmacogenomic biomarkers affecting docetaxel clearance would be of especially great value for evidence-based dose decisions. Specifically, *CYP3A4*, *CYP3A5, AAG* gene variants are known to enhance, while the *ABCC2* variant is reported to reduce docetaxel clearance in PC treatment. This may cause an elevated or reduced docetaxel dose, respectively. Docetaxel toxicity in PC treatment may be avoided by testing for polymorphisms of the following biomarker genes: *CHST3, MDR1/ABCB1, ABCC2, ABCC6, ATP7A, ATP8A2, CYP2D6, CYP4B1, GSTP1, NAT2, SLC10A2, SLCO1B3, SPG7*, and *VAC14*.

Prognostic biomarkers have a high importance from clinical and patient perspective. Better overall survival is influenced by *CYP1B1, ABCG2, MDR1, ABCB4, ABCB11, ABCC5, CYP19A1, ERα/ESR1, GSTP1* and *SLC5A6* genes. Importantly, favorable progression-free survival is related to *CYP1B1* and *VEGF-A* polymorphisms.

In summary, the most important germline pharmacogenetic biomarker originating from the research studies is *CYP1B1* rs1056836, indicating both clinical response, overall and progression-free survival. In addition, on the same way *ABCG2* rs2231142 indicates a better clinical response and overall survival. *CHST3* variants (rs4148950, rs1871450, rs4148945) indicate better clinical response and toxicity. *MDR1/ABCB1* (rs1045642, rs2032582) variants play an important role in better overall survival and toxicity, while the *ABCC2* rs12762549 variant in reduced clearance/dosing and toxicity.

Only one single clinical trial gives a hint on the use of an azacytidine demethylating agent, which can be beneficial in mCRPC patients who have increased *GADD45A* gene methylation after initial docetaxel treatment failure.

Although genetic testing is not recommended yet, these prognostic and predictive germline genomic biomarkers may have the best translational value.

## 7. Challenges, Conclusions, and Outlook

The results of the research summarized above justify the increasing number of studies aimed at identifying the associations between the genetic signatures of PC patients and docetaxel drug response, resistance, and toxicity.

However, only a minority of the significant pharmacogenetic candidates have been taken forward for clinical validation. To overcome the challenge of moving biomarkers into a clinical setting, prospective study designs, larger discovery cohorts, and subsequent clinical validation in good quality randomized trials are urgently needed.

Another challenge is how to define the best approach for biomarker selection, with enough evidence to transition them to the clinic. The hurdles include the inherent low frequency of many of these markers, the lengthy validation process through trials, and legislative and economic issues.

The predictive capacity of pharmacogenomic biomarkers for specific clinical outcome measures can be improved via composing expanded multigene pharmacogenomic panels defined by drug efficacy, drug toxicity, clinical response, or survival. Integrating these clinical effect-based pharmacogenomic panels into future research studies and clinical trials would allow a more comprehensive, evidence-based approach to determine the significance and importance of genetic testing. Furthermore, with appropriate consent and pretesting education [46], incorporating biomarker assessment provides the opportunity to not only assess cancer risk, but facilitate clinical trial eligibility and treatment selection [47]. In addition, the use of germline genomic biomarkers in cancer treatment is considered to be a less invasive approach compared to biopsy-originated somatic biomarkers.

Technological requirements for the clinical implementation of biomarker assessment are now readily available. However, it is important to ensure that continued pharmacogenetic education is provided to clinical oncologists, and that the benefit of using genetic polymorphisms as predictive biomarkers in routine and clinical research is stressed.

In summary, considerable progress has been made in the discovery of clinically applicable pharmacogenomic signatures of docetaxel treatment in PC. However, a more collaborative approach between stakeholders and studies with specific clinical output measures are needed to pave the way towards the routine use of pharmacogenomic biomarkers in personalised treatment of PC.

## Figures and Tables

**Table 2:** Completed or terminated clinical trials for docetaxel treatment of prostate cancer (ClinicalTrials.gov).

National Clinical Trial Number	Study Period	Status	Intervention	Genomic Signature	Phase	Total Number of Participants	Study Type	Results
NCT00089609	Apr 2005–Jan 2018	Completed	docetaxel + thalidomide + prednisone + bevacizumab	Association of SNPs in CYP3A4, CYP3A5 (docetaxel), and CYP2C19 (thalidomide) with pharmacokinetics and efficacy	II	73	Interventional	Yes. Association of the SNPs and efficacy was not investigated.
NCT01308567	May 2011–May 2018	Completed	cabazitaxel + prednisone or docetaxel + prednisone	Pharmacogenomics of cabazitaxel	III	1170	Interventional	Yes. Results of pharmacogenomic studies were not published.
NCT00619996	Mar 2007–Jan 2009	Completed	sorafenib + docetaxel	Gene expression profiling on blood cells and tumor biopsy	II	43	Interventional	No.
NCT00503984	May 2007–Jun 2015	Terminated (withdrawal of funding)	azacitidine + docetaxel + growth factor support	GADD45A methylation and expression after azacitidine treatment in patients whose disease is progressing on docetaxel treatment	I, II	22	Interventional	Yes. Significant demethylation of GADD45A was observed. Azacitidine may reverse docetaxel resistance.
NCT01253642	Jul 2010–Sep 2017	Terminated (low enrollment)	phenelzine sulfate + docetaxel	Frequency of MAOA overexpression CRPC tumors that are progressing on docetaxel treatment. HIF-1alpha and MAOA expression in Circulating Tumor Cells (CTCs).	II	11	Interventional	Yes. MAOA was overexpressed in all examined tumors. HIF-1alpha and MAOA expression in CTCs was not analyzed.

**Table 3:** Ongoing clinical trials for docetaxel treatment in prostate cancer (“recruiting”, “active”/”not recruiting”, “not yet recruiting”) (ClinicalTrials.gov).

National Clinical Trial Number	Status	Interventions	Genomic Signature	Phase	Participants (Estimated)	Study Type
NCT02975934	Recruiting	rucaparib or abiraterone + prednisone/enzalutamide/docetaxel + prednisone	Response in patients with evidence of a homologous recombination gene deficiency (*BRCA1*/*2* or *ATM*)	III	400	Interventional
NCT03442556	Recruiting	docetaxel + carboplatin + rucaparib	Response in patients with homologous recombination DNA repair deficiency (*BRCA1*/*2*, *ATM*, *PALB2* germline mutations)	II	20	Interventional
NCT02985021	Recruiting	docetaxel + carboplatin	Response in patients with germline or somatic inactivation of DNA repair pathway genes (*BRCA1*, *BRCA2*, *ATM*)	II	35	Interventional
NCT03517969	Recruiting	docetaxel + carboplatin or carboplatin + ATR^1^ kinase inhibitor VX-970	Response in tumors with homologous recombination deficiency	II	130	Interventional
NCT02598895	Recruiting	docetaxel + carboplatin	Response in tumors with mutation of DNA repair pathway genes (*BRCA1*, *BRCA2*, *ATM*)	NA	14	Interventional
NCT03070886	Recruiting	ADT^2^ + external beam radiotherapy + docetaxel or ADT + external beam radiotherapy	Response in genomically defined sub-groups of patients	II, III	612	Interventional
NCT02649855	Recruiting	docetaxel + PROSTVAC (vaccine)	Evaluate drug metabolism and transporters	II	74	Interventional
NCT03358563	Recruiting	ADT + docetaxel + Radical prostatectomy	Evaluation of genomic signatures and gene expression after treatment. Evaluation of biomarkers in tumor cells in circulation, as well a bone marrow before and after treatment.	Early I	30	Interventional
NCT03218826	Recruiting	docetaxel + AZD8186	Dose escalation and anti-tumor activity of AZD8186 when given together with docetaxel in patients’ solid tumors with *PTEN* or *PIK3CB* mutations. Evaluation of co-mutated genes and their association with treatment response or resistance.	I	58	Interventional
NCT02362620	Active, not recruiting	docetaxel or cabazitaxel	Exploration of prognostic biomarkers (overall survival). Evaluation of the prognostic value of *TMPRSS2-ERG* re-arrengement, *PTEN* loss, and *AR* splicing variants. Association of somatic and germline mutations and the outcomes of the patients.	NA	402	Observational (prospective)
NCT03700099	Not yet recruiting	docetaxel + enzalutamide	Association of the *AR* gene alteration, *AR-V7* status, and PSA response.	II	30	Interventional
NCT03356444	Not yet recruiting	abiraterone + prednisone or docetaxel + prednisone	Exploration of some of the genes related to the treatment efficacy	II	140	Interventional
NCT03816904	Not yet recruiting	docetaxel or paclitaxel	Determination of the number of CAG triplets in the *KCNN3/SK3* gene associated with neuropathy	NA	250	Observational (prospective)

^1^ ATR, ataxia telangiectasia and rad3-related; ^2^ ADT, androgen deprivation therapy.

**Table 4:** Clinical trials for docetaxel treatment in prostate cancer in EU Clinical Trials Register.

Eudra Clinical Trial Number	Intervention	Genomic Signature	Results	Phase/Status	Study Type/Participants	Comparison with ClinicalTrials.gov
2008-000701-11	dasatinib + docetaxel + prednisone OR placebo + docetaxel + prednisone	Samples collected for future pharmacogenomic studies	Yes. Nothing on pharmacogenomics	III/Completed	Interventional/1930	Listed on ClinicalTrials.gov Pharmacogenomic aspect was not mentioned on ClinicalTrials.gov (NCT00744497).
2007-000323-17	docetaxel + ADT (leuprolide + bicalutamide) OR ADT alone	Evaluation of gene expression profiles, genetic changes, and quantitative methylation of different genes, and their ability to predict the treatment outcome of high-risk prostate cancer subjects	Yes. Nothing on pharmacogenomics	III/Terminated	Interventional/413	Trial was listed on ClinicalTrials.gov. Pharmacogenomic aspect was mentioned in the original but not in the current secondary outcome measures on ClinicalTrials.gov (NCT00514917).
2013-000809-23	masitinib + docetaxel + prednisone OR placebo + docetaxel + prednisone	In a sub-study: relationship between genomic data and overall survival	No	III/Ongoing	Interventional/581	Trial was listed on ClinicalTrials.gov Pharmacogenomic aspect was not mentioned on ClinicalTrials.gov (NCT03761225).
2006-004478-29	docetaxel + prednisone + ciclophosphamide + celecoxib	Evaluation of the most frequent genetic polymorphisms of CYP2B6, CYP2C19, CYP2C9, and CYP3A5 and their association with the observed response	No	II/Ongoing	Interventional/45	Not found on ClinicalTrials.gov

**Table 5:** Germline genomic biomarkers in docetaxel treatment of prostate cancer with clinical translational potential.

Biomarker	Predicitive			Prognostic	
	Clinical Response (↑)	Toxicity	Dosing (Clearance)	Overall Survival (↑)	Progression Free Survival (↑)
CYP1B1 (rs1056836)	X			XXX	X
ABCG2 (rs2231142)	X			X	
CHST3 (rs4148950)	X	X			
CHST3 (rs1871450)	X	X			
CHST3 (rs4148945)	X	X			
MDR1/ABCB1 (rs1045642)		XX		X	
MDR1/ABCB1 (rs2032582)		X		X	
ABCC2 (rs12762549)		X	X (reduced)		
CHST3 (rs4148947)	X				
CHST3 (rs12418)	X				
CHST3 (rs730720)	X				
CHST3 (rs4148943)	X				
PPAR-δ (rs6922548)	X				
PPAR-δ (rs2016520)	X				
PPAR-δ (rs1883322)	X				
PPAR-δ (rs3734254)	X				
PPAR-δ (rs7769719)	X				
SULT1C2 (rs1402467)	X				
ABCC6 (rs2238472)		X			
ATP7A (rs2227291)		X			
ATP8A2 (rs11017056)		X			
ATP8A2 (rs1326116)		X			
CYP2D6*19		X			
CYP4B1 (rs4646487)		X			
GSTP1 (rs1695)		X			
NAT2 (rs1799931)		X			
SLC10A2 (rs2301159)		X			
SLCO1B3 (rs11045585)		X			
SPG7 (rs2292954)		X			
SPG7 (rs12960)		X			
VAC14 (rs875858)		X			
AAG (rs250242)			(enhanced)		
CYP3A4 (rs2740574)			X (enhanced)		
CYP3A5 (rs776746)			X (enhanced)		
ABCB4 (rs2302387)				X	
ABCB11 (rs7602171)				X	
ABCC5 (rs939336)				X	
CYP1B1 (rs1800440)				X	
CYP19A1 (rs700519)				X	
ERα/ESR1 (rs2234693)				X	
ERα/ESR1 (rs9340799)				X	
GSTP1 (rs1799811)				X	
MDR1/ABCB1 (rs1128503)				X	
SLC5A6 (rs1395)				X	
VEGF-A (rs699947)					X
VEGF-A (rs1570360)					X
VEGF-A (rs2010963)					X
VEGF-A (rs3025039)					X

## Supplementary Materials

The following are available online at https://www.mdpi.com/2073-4425/10/8/599/s1, Table S1: Withdrawn trials and trials with unknown status for docetaxel treatment in prostate cancer (ClinicalTrials.gov).
