# Supplementary material for: Pharmacogenomic Biomarkers in Docetaxel Treatment of Prostate Cancer: From Discovery to Implementation
Source: Genes (Basel). 2019 Aug 8;10(8):599. doi: 10.3390/genes10080599 (PMC6723793; doi:10.3390/genes10080599)
Supplement: Supplementary file 1 [file genes-10-00599-s001.pdf]

Supplementary table 1. Withdrawn clinical trials and clinical trials with unknown status for docetaxel treatment in prostate cancer (ClinicalTrials.gov)

NCT Number	Title	Status	Results	Interventions	Pharmacogenomic analyses	Phase	Participants	Study Type
NCT00598858	Neoadjuvant Docetaxel on Newly Diagnosed Intermediate and High Grade Cancer of the Prostate	Withdrawn (lack of funding)	No	docetaxel + prednisone	Genomic factors affecting responses to chemotherapy.	II	0	Interventional
NCT01087580	Docetaxel + Prednisone With or Without Radiation for Castrate Resistant Prostate Cancer	Withdrawn	No	docetaxel + prednisone or docetaxel + prednisone + radiotherapy	Identification of genes important in governing the response to chemotherapy and radiation therapy. Expression and mutation of p53 and AR.	II	0	Interventional
NCT02793219	Provenge Followed by Docetaxel in Castration-Resistant Prostate Cancer	Withdrawn (company decided not to fund)	No	sipuleucel-T + docetaxel	Association of immunological biomarkers with clinical results for therapy. Expression of CD3, CD4, CD8, CD25/FOX3P, CD56, CTLA-4, PD-1, and Ki67 in prostate cancer infiltrates.	II	0	Interventional
NCT02793765	Docetaxel Followed by Provenge in Metastatic Prostate Cancer	Withdrawn (company decided not to fund)	No	docetaxel + sipuleucel-T	Association of immunological biomarkers with clinical results for therapy. Expression of CD3, CD4, CD8, CD25/FOX3P, CD56, CTLA-4, PD-1, and Ki67 in prostate cancer infiltrates.	II	0	Interventional

NCT00104715	Hormone Therapy and Docetaxel or Hormone Therapy Alone in Treating Patients With Metastatic Prostate Cancer	Unknown status	No	hormonal therapy + docetaxel or hormonal therapy alone	Tumor profiles of gene expression	III	378 (estimated)	Interventional
NCT02208583	Molecular Phenotype Changes and Personalized Treatment for CRPC	Unknown status	No	docetaxel + prednisone + targeted drugs or cisplatin + etoposide + targeted drugs	Molecular phenotypic changes after acquired ADT resistance and their effect on OS (AR,Ki-67, CD56, Syn, P53, AURKA, N-myc, retinoblastoma susceptibility, E-cadherin, vimentin, hotspot mutation for 48 cancer related genes)	NA	150 (estimated)	Interventional

AR, androgen receptor
ADT, androgen deprivation therapy
OS, overall survival
